# Boosting the Anticancer Activity of *Aspergillus flavus* “endophyte of Jojoba” Taxol via Conjugation with Gold Nanoparticles Mediated by γ-Irradiation

**DOI:** 10.1007/s12010-022-03906-8

**Published:** 2022-04-19

**Authors:** Sobhy S. Abdel-Fatah, Gamal M. El-Sherbiny, Mahmoud khalaf, Ashraf F. El Baz, Ashraf S. A. El-Sayed, Ahmed I. El-Batal

**Affiliations:** 1 Drug Radiation Research Department, Biotechnology Division, National Center for Radiation Research and Technology (NCRRT), Atomic Energy Authority, Cairo, Egypt; 2 Botany and Microbiology Department, Faculty of Science (Boys), Al-Azhar University, Cairo, Egypt; 3 Microbiology Department, Biotechnology Division, National Center for Radiation Research and Technology (NCRRT), Atomic Energy Authority, Cairo, Egypt; 4 Genetic Engineering and Biotechnology Research Institute (GEBRI), University of Sadat University City, Sadat City, Egypt; 5 Enzymology and Fungal Biotechnology Lab (EFBL), Botany and Microbiology Department, Faculty of Science, Zagazig University, Zagazig, 44519 Egypt

**Keywords:** *Aspergillus flavus*, Jojoba, Endophytic fungi, Gold nanoparticles, Taxol, γ-Irradiation, Nutritional optimization

## Abstract

Taxol production by fungi is one of the promising alternative approaches, regarding to the natural and semisynthetic sources; however, the lower yield and rapid loss of Taxol productivity by fungi are the major challenges that halt their further industrial implementation. Thus, searching for fungal isolates with affordable Taxol-production stability, in addition to enhance its anticancer activity via conjugation with gold nanoparticles, is the main objectives of this study. Twenty-four endophytic fungal isolates were recovered from the barks, twigs, and leaves of jojoba plant, among these fungi, *Aspergillus flavus* MW485934.1 was the most potent Taxol producer (88.6 µg/l). The chemical identity of the extracted Taxol of *A. flavus* was verified by the TLC, HPLC, HNMR, and FTIR analyses. The yield of Taxol produced by *A. flavus* was optimized by the response surface methodology (RSM) using Plackett–Burman (PBD) and faced central composite designs (FCCD). The yield of Taxol by *A. flavus* was increased by about 3.2 folds comparing to the control cultures (from 96.5 into 302.7 µg/l). The highest Taxol yield by was obtained growing *A. flavus* on a modified malt extract medium (g/l) (malt extract 20.0, peptone 2.0, sucrose 20.0, soytone 2.0, cysteine 0.5, glutamine 0.5, and beef extract 1.0 adjusted to pH 6.0) and incubated at 30 °C for 16 days. From the FCCD design, the significant variables affecting Taxol production by *A. flavus* were cysteine, pH, and incubation time. Upon *A. flavus* γ-irradiation at 1.0 kGy, the Taxol yield was increased by about 1.25 fold (375.9 µg/l). To boost its anticancer activity, the purified Taxol was conjugated with gold nanoparticles (AuNPs) mediated by γ-rays irradiation (0.5 kGy), and the physicochemical properties of Taxol-AuNPs composite were evaluated by UV–Vis, DLS, XRD, and TEM analyses. The IC_50_ values of the native-Taxol and Taxol-AuNPs conjugates towards HEPG-2 cells were 4.06 and 2.1 µg/ml, while the IC_50_ values against MCF-7 were 6.07 and 3.3 µg/ml, respectively. Thus, the anticancer activity of Taxol-AuNPs composite was increased by 2 folds comparing to the native Taxol towards HEPG-2 and MCF-7 cell lines. Also, the antimicrobial activity of Taxol against the multidrug resistant bacteria was dramatically increased upon conjugation with AuNPs comparing to authentic AuNPs and Taxol, ensuring the higher solubility, targetability, and efficiency of Taxol upon AuNPs conjugation.

**Supplementary Information:**

The online version contains supplementary material available at 10.1007/s12010-022-03906-8.

## Introduction

Taxol is one of the most commercialized broad spectrum anticancer drugs [1]. The activity of Taxol elaborates from its unique specificity for binding with the cellular tubulin β-subunits heterodimer, promoting tubulin polymerization, thus disrupting the mitotic division of tumor cells [2]. Taxol displayed a strong activity against breast, lung, head and neck, uterine cancers, and advanced forms of Kaposi’s sarcoma [3]. Taxol was firstly produced from the bark of yew trees *Taxus brevifolia* “family Taxaceae” [4, 5]; however, the lower yield of Taxol that being < 0.001%, i.e., to produce 1 g Taxol, it requires ~ 10 kg of plant bark that collected from 3 to 5 trees [6], is the main challenge. In addition, the vulnerability of this plant to unpredicted fluctuations with the environmental conditions strongly influences the Taxol yield, heterogeneity, and reproducibility [7–9]. Exploring the Taxol producing potency of the endophytic fungi inhabiting medicinal plants raises the hope for overcoming the low yield by the above-mentioned method [10, 11], due to their fast growth, cost-effectiveness, independence on climatic changes, and feasibility for genetic manipulation [12, 13]. Subsequently, a plethora of endophytic fungi with metabolic potency to produce Taxol has been reported as reviewed [1, 14–25]. However, the anticipation of these fungi for industrial production of Taxol has been challenged by the attenuation and loss of Taxol productivity by the fungal storage and multiple subculturing [21, 22, 26–28].

Thus, searching for a novel fungal isolate with affordable metabolic stability and sustainability for Taxol production is the challenge. Medicinal plants of well-known ethnopharmacological relevance and traditional pharmaceutical applications could be the repertoire of novel fungal isolates with unique features of metabolic stability for Taxol biosynthesis. Among the most common medicinal plants, jojoba “*Simmondsia chinensis*” is a monogenetic dioecious grey-green shrub belonging to Simmondsiaceae family. Jojoba seeds contain up to 65% of a light golden and odorless high-viscosity oily metabolites [29]. Jojoba oil has been frequently used for the relief of headaches, throat inflammation, and wounds treatment [30, 31]. As well as Jojoba oil has been used as anti-inflammatory and antimicrobial agents [30, 31]. The leaves of jojoba are rich with antioxidant flavonoid compounds that traditionally used for treating of various disorders such as asthma, inflammation, and cancer [32]. Thus, the main objective of this work was to explore a new fungal isolate from jojoba plant with unique metabolic stability for Taxol production, to evaluate the different approaches to maximize their Taxol yield, as well as, to enhance the antiproliferative activity of extracted Taxol compounds via conjugation with gold nanoparticles, mediated by gamma irradiation.

## Material and Methods

### Isolation and Culturing of the Endophytic Fungi

Different parts of jojoba (*Simmondsia chinensis*) as leaves, barks, twigs, and buds were collected from Faculty of Agriculture, Cairo University and used as the source for endophytic fungi. The plant parts were collected and washed under running tap water, surface sterilized with 70% ethanol for 1 min and then rinsed with sterile water [28]. The surface-sterilized plant parts were cut into small pieces under sterile condition and placed on plates of potato dextrose agar (PDA) medium, Czapek’s-Dox, and malt extract agar medium [33–36], and the plates were incubated at 30 °C for 10 days. The effectiveness of surface sterilization of the plant parts was assessed by centrifuging the rinsing water, then 500 μl sterile water was added to the precipitate and plated into PDA medium [37]. The purified endophytic fungal isolates were inoculated on PDA slants for 7 days and stored at 4 °C.

### Screening, Extraction, and Quantification of Taxol from the Endophytic Fungi

The recovered endophytic fungi inhabiting jojoba were screened for Taxol production by growing on potato dextrose broth (PDB) [38]. One plug of each of the 7 days old fungal isolates was inoculated into 100 ml of PDB/250 ml Erlenmeyer flasks, incubated for 15 days at 30 ± 1 °C, under shaking conditions (120 rpm). After incubation, the cultures were filtered, and the filtrates were amended with 0.2% sodium bicarbonate to precipitate fatty acids. Taxol has been extracted with dichloromethane, and the organic phase was collected and evaporated to dryness, and the residues were re-dissolved in methanol [17, 39]. Taxol was separated and identified by TLC using Merck 1 mm (20 × 20 cm) pre-coated silica gel plates (TLC Silica gel 60 F254, Darmstadt, Germany), detected by UV illumination at 254 nm [39]. The putative spots of Taxol were scraped-off from the TLC silica gel plates and dissolved in methanol, vortexed vigorously for 10 min, and centrifuged at 1000 rpm for 5 min. The precipitated silica particles were removed, and the supernatant was taken for Taxol quantification and purity checking by HPLC (YOUNG In, Chromass, 9110 + Quaternary Pump, Korea) of C18 reverse phase column (Eclipse Plus C18 4.6 × 150 mm, 3.5 μm, Cat. # 959,963–902). The mobile phase used was methanol/acetonitrile/water (25:35:40, v/v/v) at a flow rate of 1.0 ml/min for 20 min [40], and Taxol fractions were measured at 227 nm, and their chemical identity and concentrations were confirmed from the retention time and absorption peak area comparing to authentic sample.

### Morphological and Molecular Identification of the Recovered Endophytic Fungi

The endophytic fungal isolates were identified to their species levels based on their macro and micro-morphological features by growing on PDA, Czapek’s-Dox, and malt extract media according to the reference’s keys [33–36]. The identity of the most potent Taxol producing fungal isolates were further molecularly confirmed based on the sequence of internal transcribed spacer (ITS) [41, 42]. Fungal genomic DNA (gDNA) was extracted by pulverizing the mycelia (~ 0.2 g) in liquid nitrogen, then dispensing in 1 ml CTAB extraction buffer (2% CTAB, 2% PVP40, 0.2% 2-mercaptoethanol, 20 mM EDTA, 1.4 M NaCl in 100 mM Tris − HCl, pH 8.0). The PCR primer sets were ITS4 5′-GGAAGTAAAAGTCGTAACAAGG-3′ and ITS5 5′-TCCTCCGCTTATTGATATGC-3′. The PCR reaction contains 10 μl of 2 × PCR master mixture (i-Taq™, Cat. No. 25027), 2 μl of gDNA, 1 μl of each primer (10 pmol/μl), and completed to 20 μl with sterile distilled water. The PCR was programed to initial denaturation at 94 °C for 2 min, denaturation at 94 °C for 30 s, annealing at 55 °C for 10 s, extension at 72 °C for 30 s for 35 cycles, and final extension at 72 °C for 2 min. The PCR amplicons were analyzed by 1.5% agarose gel in 1 × TBE buffer (Ambion Cat# AM9864), using 1 kb DNA ladder (Cat. # PG010-55DI) and visualized by gel documentation system. The amplicons were purified and sequenced by Applied Biosystems Sequencer, HiSQV Bases, Version 6.0 with the same primers sets. The obtained sequences were BLAST searched non-redundantly on the NCBI database, imported into MEGA 6.0 software and aligned with Clustal W muscle algorithm [43] and the phylogenetic tree was constructed with neighbor-joining method of MEGA 6.0 [44].

### Chemical Structure of the Extracted Taxol

The putative spots of Taxol were scraped-off from the TLC silica gel plates, purified, and the purity and concentration were determined by the UV–Vis analyses at λ _227_ nm (RIGOL, Ultra-3000 Series) comparing to authentic Taxol [39]. Blank media under the same conditions were used as negative baseline for the spectrophotometric analyses. FT-IR spectrum of the purified Taxol samples was analyzed by JASCO FT-IR 3600 spectrophotometer. The Taxol sample was grinded with KBr pellets, pressed into discs under vacuum, and the absorption was measured in the region 400 to 4000 cm^−1^ [3], comparing to authentic one. The chemical structure of extracted Taxol was confirmed from the HNMR spectroscopy (JEOL, ECA-500II, 500 MHz NMR) comparing to authentic Taxol. The samples were dissolved in CDCl_3_, chemical shifts are given in ppm (δ-scale), and the coupling constants are expressed in hertz (Hz).

### Effect of Different Types of Media on Taxol Production

Two agar plugs (9 mm) from 7 days old cultures of each fungal isolate were inoculated in triplicate into 100 ml medium/250 ml Erlenmeyer flask of potato dextrose (PDB), Czapekʼs-Dox (CZD), M1D, and malt extract (ME) broth media. Uninoculated controls from each media free of fungal spores were used as negative control, incubated at 30 °C for 15 days under the same conditions. After incubation, fungal cultures were filtered, and Taxol was extracted and determined as mentioned above.

### Bioprocess Optimization of the Nutritional Conditions to Maximize the Taxol Yield

Optimization of the medium composition for maximizing the Taxol yield by the potent fungal isolate was conducted by response surface methodology using Placket-Burman design followed by central composite design [17–20, 45]. From the RSM designs, the positive and significant variables affecting Taxol production by the potent fungal isolate were assessed using the statistical software package by Design-Expert 7.0 (Stat Ease Inc., Minneapolis, USA). Each experiment was run in three biological replicates and the mean values were considered. After incubation at the desired conditions, fungal biomass was filtrated, and Taxol was extracted, and quantified by TLC and HPLC as described above.

#### Placket-Burman Design

Placket-Burman design has been frequently used for optimization of the media component for fungal growth and production of bioactive secondary metabolites, evaluating the significant variables affecting Taxol production [18, 20, 46]. Choice of factor was based on media used in qualitative and quantitative screening. Eleven factors have been included; malt extract, peptone, sucrose, soytone, glutamine, beef extract, and temperature, pH, incubation time, and shaking speed values and factors were varied over two levels, and the minimum and maximum levels ranges were selected. The statistical Design-Expert 7.0 was used to generate a set of 12 experiments. For each experiment, Taxol production was determined in three biological replicates, and the average of Taxol yield was considered. Regression analysis of the data was conducted using statistical software. The effect of each variable was calculated (Biometrika, 2020), using the following equation:

$$E=(\sum {M}_{+}-\sum \frac{{M}_{-}}{N})$$

where, E is the effect of a testing variable, M_+_ and M_−_ are Taxol concentration of trials at that the parameter was at its higher and lower levels respectively, and N is the number of experiments that was carried out. The effect of each variable on the production was determining by calculating their respective E-values.

$$E=\frac{{Tot}_{hi\mathrm{g}h}-{Tot}_{low}}{No}$$

Where Tot _high_ is the total responses at the high level, Tot _low_ is the total responses at the low level, and No is the number of trials.

#### Central Composite Design and Interactions Between Factors Affecting Taxol Production

The most significant positive factors affecting Taxol production by the selected fungal isolate were optimized using a response surface type CCD model experimental design [47]. By using CCD, the concentrations of the medium components were optimized, and their studied interactions were used to generate a total of 20 experiments for the three variables.

To determine the optimal levels of the variables for Taxol production from the potent fungal isolate, three-dimensional (3D) response surface curves were plotted to study the interaction between the various factors, and to determine the variable condition of each factor affecting Taxol production. The 3D graphs were carried out by holding three factors’ constants in an ideal level and plotting the obtained response of Taxol yield for varying levels of the other two factors.

### Effect of Gamma Irradiation on Taxol Yield

The potent endophytic isolates producing Taxol were exposed to γ-irradiation with ^60^Cobalt source (Gamma cell 4000-A-India) at different gamma radiation doses (0.25–3.0 kGy) compared to the non-irradiated cultures control; a dose rate 1.2 kGy/h at the time of experiments. The optimized media were inoculated by the irradiated cultural under standard cultural conditions, compared to the non-irradiated spore’s inoculum as control. The cultures were incubated at 30 ± 2 °C for 15 days on a rotary shaker (120 rpm). After incubation, the cultures were filtered and Taxol was extracted, purified, and quantified by TLC and HPLC as described above.

### Synthesis and Characterization of Gold Nanoparticles (AuNPs); Conjugation with Taxol

Polyvinylpyrrolidone (PVP)-capped gold nanoparticles (AuNPs) were synthesized by mixing 1 mM PVP (dissolved in distilled water) with 0.5 mM gold (III) chloride hydrate magnetically stirred, and the solution was irradiated by gamma rays at different doses (0.25–10.0 kGy). The obtained PVP-Au^3+^ solution has been amended with 1 ml sodium borohydride (1 mM) as reducing agent. Taxol (100 µg/ml) was mixed with PVP-AuNPS at ratio 1:2 (v/v), and the obtained Taxol-PVP-AuNPs conjugate was characterized by the UV–Vis analysis.

The size distribution and average particle size of the Taxol-PVP-AuNPs conjugate was measured by dynamic light scattering (DLS) (PSS-NICOMP 380-ZLS particle sizing system St. Barbara, CA, USA, at NCRRT). FTIR measurements were carried out to obtain information about chemical groups of the Taxol-PVP-AuNP conjugates in relation to their structural stability, comparing to the native Taxol (JASCO FT-IR 3600 infra-red spectrometer). The size and morphology of the synthesized AuNPs were recorded by using high-resolution transmission electron microscope (HRTEM), and drop coating AuNPs prepared TEM studies onto carbon-coated TEM grids. The X-ray diffraction (XRD) patterns were obtained with the XRD-6000 series, including residual austenite quantitation, stress analysis, crystallinity calculation, and crystallite size/lattice strain materials analysis by overlaying X-ray diffraction patterns (Shimadzu apparatus with Cu-Kα target, and nickel filter Shimadzu Scientific Instruments (SSI), NCRRT).

### Anticancer Activity of Taxol

The activity of the purified Taxol and Taxol-PVP-AuNPs conjugates against liver carcinoma (HPG2), and breast carcinoma (MCF7) was determined by 3- (4,5-dimethylthiazol-2-yl)-2,5-diphenyl tetrazolium bromide (MTT) assay [48]. The 96-well plate was seeded with 10^3^ cells per well, incubated overnight at 37 °C, then different concentrations of the drug were added, and the plates were re-incubated for 48 h. The MTT reagent (25 μl) was added, incubated for 2 h, and the purple color of the developed formazan complex was measured at λ570 nm. The IC_50_ value was expressed by amount of drug reducing the growth of 50% of initial number of tumor cells normalizing to positive control.

### Antimicrobial Activity of Taxol and Taxol-AuNPs Conjugates

The antimicrobial activity of Taxol and Taxol-AuNPs conjugates was assessed against different bacterial isolates; *Bacillus subtilis* ATCC 6633 and *Staphylococcus epidermidis*, *Pseudomonas aeruginosa*, *Escherichia coli*, and *Enterobacter agglomerans*, in addition to *Candida albicans*. The tested bacterial cells were suspended in sterile peptone water to obtained standard inoculum of ~ 0.5 McFarland (1–1.5) × 10^8^ CFU/ ml at λ_600_ nm. The growth inhibition (mm) of microbial pathogens growth was assessed by agar disc diffusion method. Sterile standard antibiotic disks with the diameter of 6.0 mm were used as positive controls. Sterile antibiotic discs (6.0 mm) were loaded with 20 μl of methanol and amoxicillin clavulanic acid (AMC) as negative and positive control. Discs were loaded with the same concentration of Taxol, Taxol-PVP-AuNPs, and AuNPs (1.0 μg/ml). Three biological replicates were prepared. The plates were incubated at 37 °C for 24 h, and the zones of inhibition were measured. Amoxicillin clavulanic acid (AMC) and nystatin were used to normalize the antimicrobial activity of Taxol. The inhibition zone of growth was determined by a vernier caliper (mm).

### Statistical Analyses

The experiments were conducted in three biological replicates, and the results were expressed by mean ± STDV. The significance was calculated by one-way ANOVA with Fisher’s least significant difference of post hoc test (https://www.easycalculation.com/statistics/fishers-lsd-calculator.php).

### Fungal Deposition

The isolate *A. flavus* Bd was deposited at genbank under accession #MW485934.1 as well as at Assiut University Mycological Center (AUMC), Egypt, with deposition #AUMC13892.

## Results

### Isolation of Endophytic Fungi from Jojoba; Screening for Taxol Production

**Fig. 1:**
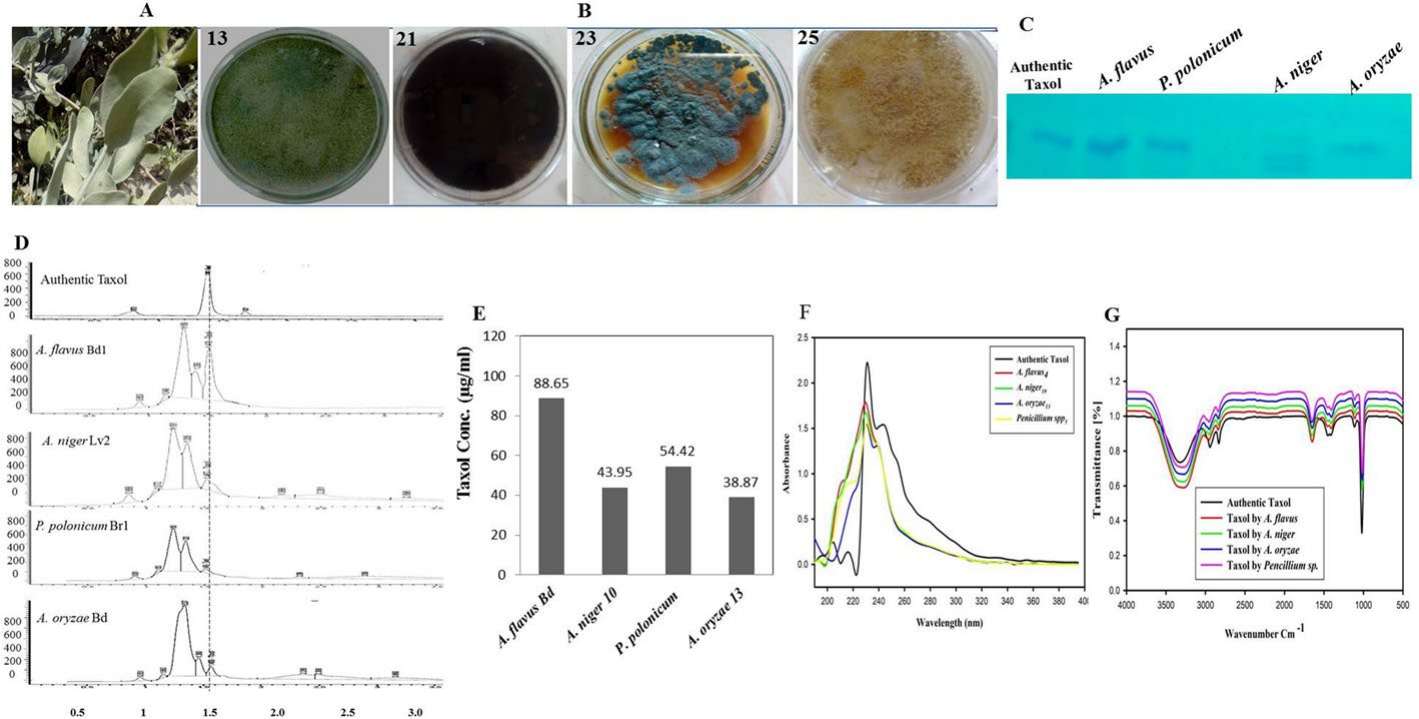
**A** Morphological views of jojoba plant. **B** Plate cultures of the potent Taxol producing endophytic fungi; *A. flavus* Bd1 (13), *A. niger* Lv1 (21), *Penicillium polonicum* (23), and *A. oryzae* Bd (25) on PDA after 8 days o incubation at 30 °C. The fungal isolates were grown on PDB, incubated at the standard conditions, and Taxol was extracted and checked by TLC (**C**). **D** HPLC chromatogram of Taxol from the potent fungal isolates. **E** Yield of Taxol as quantified from HPLC. F, UV–Vis spectral analysis of extracted Taxol from the fungal isolates. **G** FT-IR analysis of extracted Taxol comparing to authentic one

Twenty-four endophytic fungal isolates were recovered from the barks, twigs, leaves, and buds of jojoba loaded on PDA, CZD, and ME medium. These fungal isolates were derived from barks (6 isolates), twigs (7 isolates), leaves (4 isolates), and buds (7 isolates) as recorded in Table 1. These fungal isolates were initially identified to their species level based on their morphological features according to the universal keys, belonging to three genera, namely *Aspergillus*, *Penicillium*, and *Fusarium*. Among these isolates, the prevalence of genus *Aspergillus* was reported to be (83.4%), while *Fusarium* and *Penicillium* were represented by 8.3%. The genus *Aspergillus* was represented by five species, namely *A. flavus* (3 isolate), *Aspergillus oryzae* (5 isolates), *A. niger* (5 isolates), *A. fumigatus* (4 isolate), and *A. terreus* (3 isolates). The productivity of Taxol by the recovered fungal isolates was assessed by growing on PDB, incubation at the standard conditions, extraction, and quantification of Taxol by TLC and HPLC (Fig. 1). From the results, the maximum Taxol productivity was reported by *A. flavus* Bd1 (88.65 µg/l), followed by *P. polonicum* Br1 (54.42 µg/l), *A. niger* Lv1 (43.95 µg/l), *A. oryzae* Bd1 (38.87 µg/l), *F. oxysporum* Tw1 (26.80 µg/l), *A. niger* Lv2 (23.01 µg/l), and *A. fumigatus* Bd2 (17.62 µg/l). The structural chemical identity of Taxol from the highest fungal producers was revealed from their UV–Vis spectra, compared to the chemical spectral of the authentic Taxol. Additionally, the chemical structure of Taxol extracted from the most potent four fungal isolates has been validated by FT-IR analyses (Fig. 1). Remarkably, the extracted Taxol from the potent fungal isolates displayed the same spectral paradigm of authentic Taxol. The peak at 3393.3 cm^−1^ was assigned for the hydroxyl (OH). While the peaks at 2923.5 were assigned to the aliphatic CH stretch, the peaks at 1661.0 cm^−1^ corresponds to C = O stretching frequency. The observed peak at 1452.0–1404.0 cm^−1^ was due to the NH stretching frequency. The carbonyl group-oxygen stretching frequency was observed at 1109 cm^−1^. The observed peaks in the range 1020–979.7 cm^−1^ were due to the presence of aromatic C and H bends. From the chromatographic and spectral analyses, it could be concluded that the extracted Taxol is identical to the authentic one. Apparently, the metabolic activity of the same fungal species was greatly fluctuated with the different plant, ensuring the unique biological interaction and release of specific signals from the plant part to triggers the expression of machinery system of Taxol biosynthesis. Interestingly, the fluctuation on the metabolic system is not only dependent on the plant parts but also on isolate-isolate interaction, for example, the Taxol yield of *A. niger* isolate inhabiting the leaves of jojoba was 43.9 µg/l, while the yield of Taxol was zero for the *A. niger* isolate from recovered from the plant bark.

**Table 1:** Screening for Taxol producing endophytic fungi of *jojoba*

**Plant parts**	**Isolate no**	**Isolate code**	**Fungal isolate**	TLC spot density	HPLC (μg/L)
**Bark**	1	-	*Aspergillus niger*	-	-
	2	15	*Aspergillus terreus*	+	15.35
	3	16	*Fusarium* spp.	+	11.83
	4	22	*Aspergillus niger*	+	13.51
	**5**	-	*Aspergillus fumigatus*	-	-
	6	23	*Penicillium polonium* Br1	+ +	54.42
**Twigs**	7	-	*Aspergillus fumigatus*	-	-
	8	17	*Aspergillus oryzae*	+	15.55
	9	-	*Aspergillus niger*	-	-
	10	-	*Penicillium* sp*.*	-	-
	11	-	*Aspergillus terreus*	-	-
	12	24	*Fusarium oxysporum* Tw1	+	26.80
	13	-	*Aspergillus oryzae*	-	-
**Leaves**	14	18	*Aspergillus niger* Lv2	+	23.01
	15	-	*Aspergillus terreus*	-	-
	16	25	*Aspergillus niger* Lv1	+ +	43.95
	17	-	*Aspergillus oryzae*	-	-
**Buds**	18	13	*Aspergillus flavus* Bd1	+ + +	88.65
	19	19	*Aspergillus parasiticus* Bd2	+	15.56
	20	20	*Aspergillus ustus* Bd3	+	14.15
	21	21	*Aspergillus fumigatus* Bd1	+	14.48
	22	26	*Aspergillus fumigatus* Bd2	+	17.62
	23	-	*Aspergillus oryzae* Bd1	+ +	38.87
	24	27	*Aspergillus oryzae* Bd2	-	-

### Morphological and Molecular Identification of the Potent Taxol Producers

**Fig. 2:**
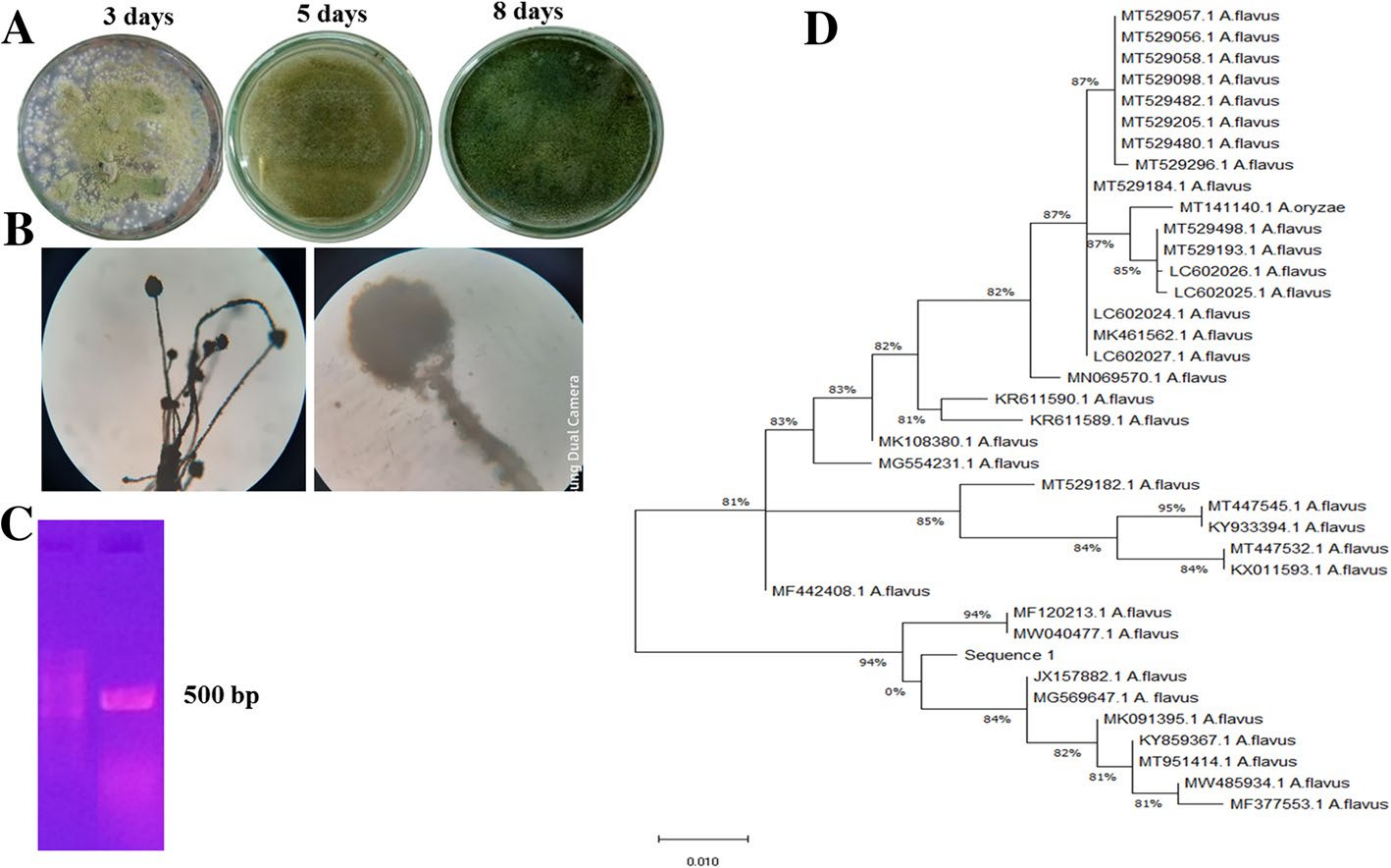
**A** Macromorphological features of *A. flavus* an endophyte of jojoba after 3, 5, and 8 days of growth on PDA. Micro-morphological features, conidial head of *A. flavus* by 400X magnification. **C** PCR amplicon of *A. flavus* ITS region of 500 bp, normalizing to 1 kb ladder (Cat.#. SM0312). **D** Phylogenetic analysis of ITS *A. flavus* by maximum likelihood method [44]

Morphological features of the potent fungal isolate producing Taxol were examined according to the macroscopical and microscopical descriptive keys, as described in Materials and Methods, and revealed its morphological proximity with *A. flavus* (Fig. 2). The fungal isolate was grown on PDA at 30 °C for 10 days and the macroscopical and microscopical features revealed its identity to such as conidial heads, mode of branching, identity of strigma, and conidial ontology, and fruiting bodies formation, according to the universal morphological keys [33], and were found to be identical to *Aspergillus flavus*. The potent Taxol producing isolate *A. flavus* was further identified based on their ITS sequences, using gDNA as template. The PCR amplicons (~ 550 bp) of *A. flavus* were resolved, purified, and sequenced (Fig. 2). The ITS sequence of *A. flavus* was non-redundant blast searched on NCBI database, displaying 99% similarity with *A. flavus*, with zero *E.* values, and 95% query coverage. Thus, from the microscopical and molecular analyses, the target isolate was confirmed as *A. flavus* and deposited on GenBank with accession number MW485934.1, as well as, the isolate has been deposited at Assiut University Mycological Center (AUMC), Egypt with a deposition number AUMC13892. The current isolate had 99% similarity with *A. flavus* isolates MW485934, MT446145, KJ863514, MW522551, MK108386, KY926854, MK091395, MG554231, KY859367, JX157882, LC6020227, LC602024, KR611590, MK461562, JX912560, MT447545, and MT447532, with zero E. value and 95% query coverage. The phylogenetic relatedness of *A. flavus* with the database deposited isolates was constructed (Fig. 2). Based on the ITS sequence, three phylogenetic clades of *A. flavus* were recovered with a strong sequence similarity as revealed from the root value 0.001, the target isolate belongs to *A. flavus* clade I.

### Chemical Identity and Anticancer Activity of A. flavus Taxol

**Fig. 3:**
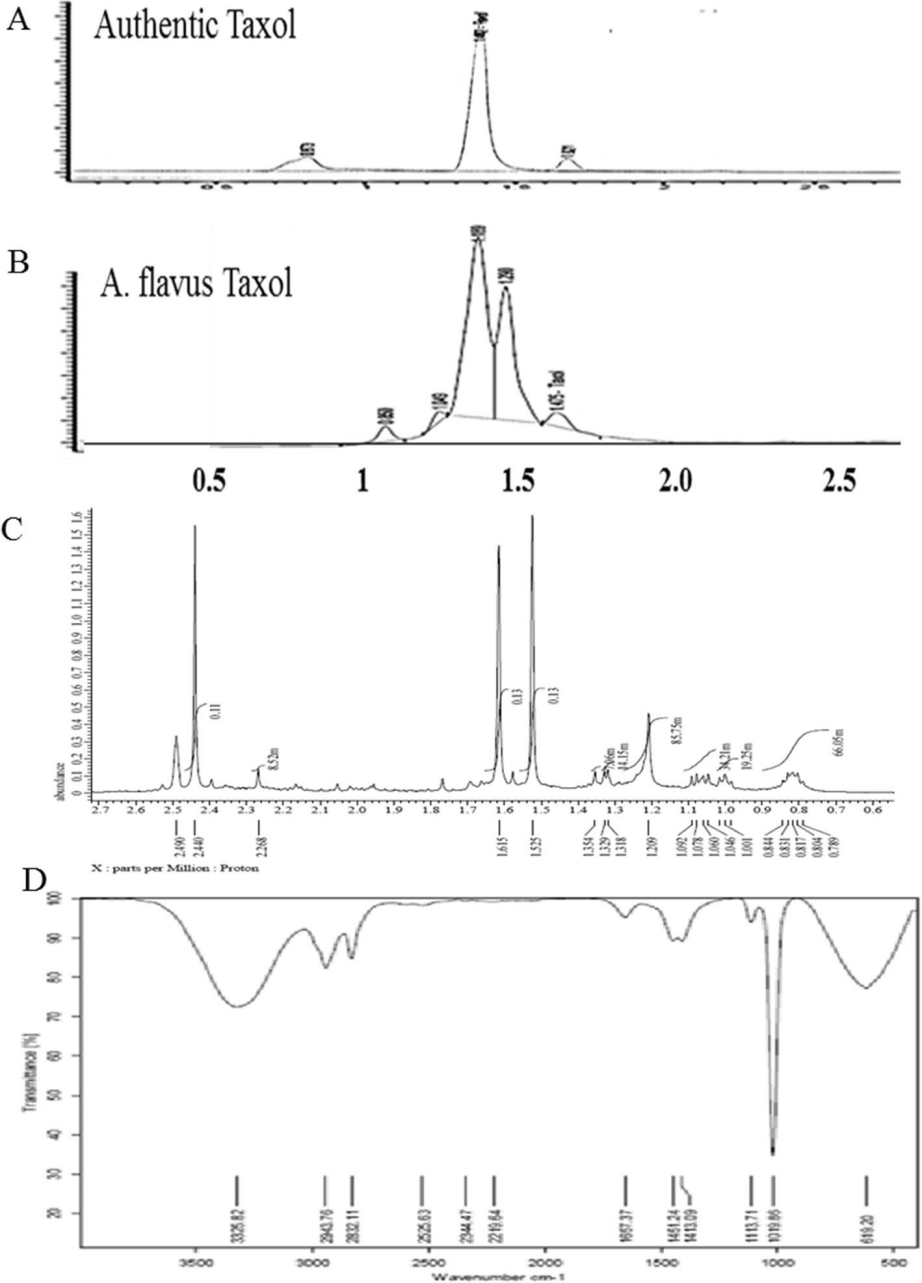
Chemical analysis of extracted Taxol from *A. flavus*. Taxol was extracted, fractionated by TLC. The putative spots of Taxol were scraped-off from the TLC plates and analyzed. HPLC chromatogram of authentic Taxol (**A**), *A. flavus* Taxol (**B**). **C** Spectra of 1HNMR of *A. flavus* Taxol sample. **D** FT-IR spectra of extracted Taxol of *A. flavus*

The putative TLC-spots of *A. flavus* Taxol were scrapped-off and dissolved in methanol, and the chemical properties of the compound were resolved from the different HPLC, HNMR, and FT-IR chromatographic and spectroscopic analyses. Proton nuclear magnetic resonance signals (^1^H NMR) of *A. flavus* extracted Taxol were distributed between 1.0 and 8.0 ppm, with three resolved proton signals at 1.0–3.0 ppm corresponding to methyl, acetate, and acetylene groups, whereas proton signals at 6.5–9.0 ppm corresponding to the aromatic moieties (Fig. 3). The pattern and distribution of protons signals of *A. flavus* Taxol authenticate the structural identity and similarity of sample with the standard Taxol (Fig. 3). Moreover, Taxol of *A. flavus* has the same FTIR spectra of authentic Taxol. The peak at 3393.3 cm^−1^ was assigned for the hydroxyl (OH), while the peak at 2923.5 assigned to the aliphatic CH stretch, the peaks at 1661.0 cm^−1^ corresponding to C = O stretching frequency. The observed peaks at 1452.0 cm^−1^ and 1404.0 were referenced to the NH stretching frequency, while the peak at 1109.0 cm^−1^ refers to the carbonyl group-oxygen stretching frequency. The peaks in the range between 1020.0 and 979.77 cm^−1^ were referenced to the presence of aromatic C and H bends. Conclusively, the spectral pattern of FTIR regarding to the functional groups of *A. flavus* Taxol was identical to the authentic Taxol (Fig. 3). Conclusively, from the chromatographic and spectral analyses, the putative sample has been resolved and authenticated as Taxol compounds comparing to the authentic compound.

### Effect of Different Types of Medium on Taxol Production by A. flavus

**Fig. 4:**
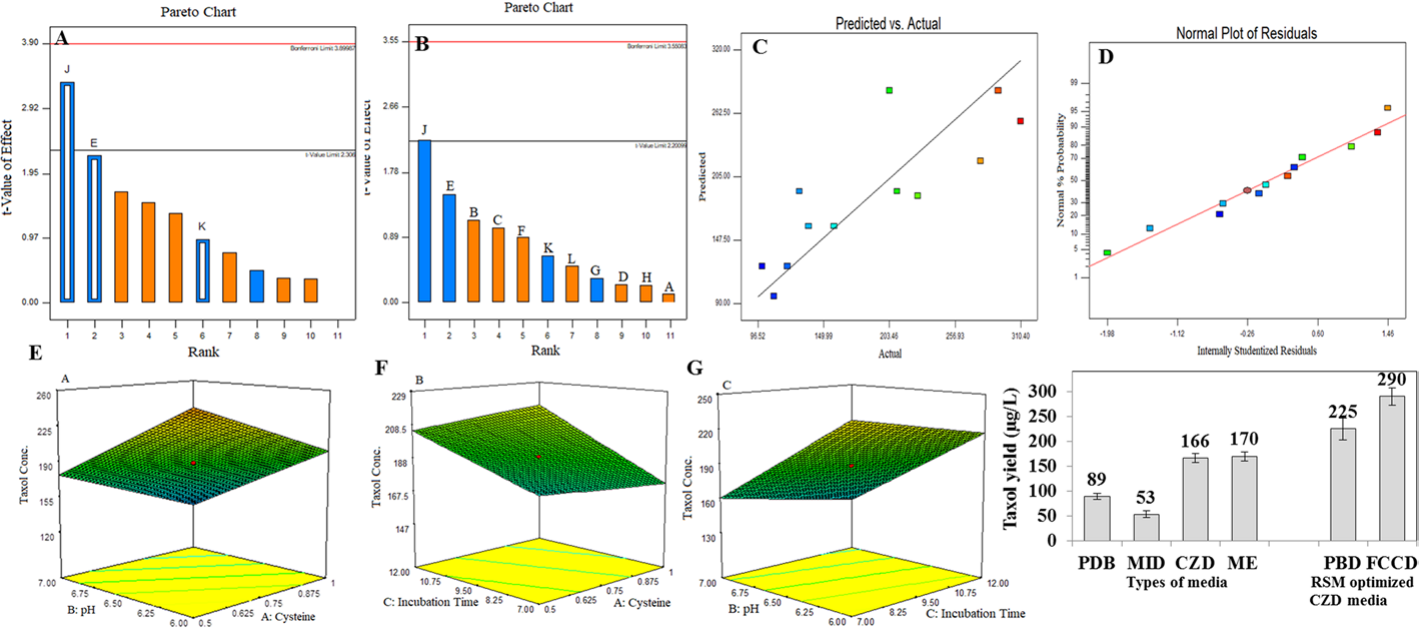
Nutritional optimization of Taxol production from *A. flavus* using Plackett–Burman design. A and B Pareto charts showing the effect of individual factors on Taxol production (A malt extract, B peptone, C sucrose, D soytone, E cysteine, F glutamine, G beef extract, H temperature, J pH, K incubation time, and L shaking speed. C Normal probability plots of the variables for Taxol production by *A. flavus* from the first order polynomial equation. D Plot of correlation between predicted and actual Taxol yield by *A. flavus*. Three-dimensional response surface curves showing the effect of interactions of pH and cysteine (E), incubation time and cysteine (F), and pH and incubation time (G)

The influence of medium composition on the Taxol production by *A. flavus* has been evaluated by growing on PDB, M1D, Czapek’s-Dox, and malt extract. After cultural incubation, Taxol was extracted and quantified as described in Materials and Methods. From the results in Fig. 4, the highest Taxol yield by *A. flavus* was obtained by growing on malt extract (170 µg/l) and Czapek’s-Dox (166 µg/l), compared to PDB growth medium as control (89 µg/l). Thus, the yield of Taxol by *A. flavus* was increased by approximately 2 folds by its growing on Czapek’s-Dox and malt extract media, compared to the control medium. So, further optimization studies to maximize the Taxol yield by *A. flavus* by the response surface methodology statistical approach have been implemented.

### Optimization of Bioprocess Variables Using Response Surface Methodology

#### Plackett–Burman Design Screening for Critical Factors

The yield of Taxol by *A. flavus* has been optimized by the statistical experimental design of Plackett–Burman [18, 28]. The minimum and maximum ranges of the selected parameters selected by Plackett–Burman design were summarized in Table 2. Taxol concentration was determined by the TLC and HPLC. All the experiments were performed in triplicate and the average Taxol concentration was considered. Statistical analysis of this design illustrates that the model F-value of 5.98 implies the model is significant and values of “Prob > F” less than 0.0500 as reported in Table 3. The actual and predicted yield of Taxol in response to the experimental parameters as revealed from the Plackett–Burman design was shown from the matrix in Table 4. The “Pred R-Squared” of 0.3061 is not as close to the “Adj R-Squared” of 0.5760 as one might normally expect. The maximum actual (255.35 µg/l) and predicted (280.4 µg/l) yield of Taxol by *A. flavus* upon factorial design by Plackett–Burman design was obtained at run # 8. From the obtained data (Fig. 4), the highest Taxol yield by *A. flavus* (225 µg/l) was obtained using malt extract (20 g/l), peptone (2 g /l), sucrose (20 g /l), soytone (2 g/l), cysteine (0.5 g/l), glutamine (0.5 g/l), beef extract (1 g/l), at incubation temperature 30 °C, initial pH 6.0, and after 12 days of incubation at shaking speed (150 rpm). At this cultural run (12), the yield of Taxol by *A. flavus* was significantly increased by about 1.4 folds, comparing to the control medium (170 µg/l). The model reduction, response tranformation, and Adeq Precision measure the signal to noise ratio, and the ratio greater than 4 is desirable [46]. Based on the parameter estimated and by applying multiple regression analysis on the experimental data, the test variables and the response variable were relating by the following model equation: first-order equation in terms of coded factors — Taxol Con. =  + 11.51 + 1.91E + 2.92 J + 0.81 K. The actual and predicted levels of Taxol by *A. flavus* upon Plackett–Burman optimization bioprocess are summarized in Table 4 and Fig. 4. The maximum actual (255.35 µg/l) and predicted (280.4 µg/l) yield of Taxol by *A. flavus* upon factorial design by Plackett–Burman design was obtained at run # 8 at medium component. The effect of individual parameters as revealed from the Plackett–Burman design was represented in Pareto Chart depicting the order of significance of variables involved in Taxol production Fig. 4. Thus, the most significant variables affecting Taxol productivity by *A. flavus* were cysteine, pH, and incubation time as revealed from the Plackett–Burman design.

**Table 2:** Minimum and maximum ranges of the parameters selected in P-BD for optimization of Taxol production

No	Factors	Levels	
		Minimum	Maximum
**1**	Malt extract (g /L)	20.00	40.00
**2**	Peptone (g /L)	1.00	2.00
**3**	Sucrose (g /L)	10.00	20.00
**4**	Soytone (g/L)	1.00	2.00
**5**	Cysteine (g/L)	0.50	1.00
**6**	Glutamine (g/L)	0.50	1.00
**7**	Beef extract (g/L)	1.00	2.00
**8**	Temperature (°C)	25.00	30.00
**9**	pH	6.00	7.00
**10**	Incubation time (days)	7.00	12.00
**11**	Shaking speed (rpm)	120.00	150.00

**Table 3:** Analysis of variance (ANOVA) for Taxol production concentration

Source	Sum of squares	df	Mean square	*F* value	*p*-value Prob > F	Significance	R-squared	Std. dev
Model	153.79	3	51.26	5.98	0.0193	Significant	0.6916	2.93
E-cysteine	43.59	1	43.59	5.08	0.0541	Significant		
J-pH	102.38	1	102.38	11.94	0.0086	Significant		
K-incubation time	7.82	1	7.82	0.91	0.3673	Significant		
Residual	68.58	8	8.57					
Cor total	222.36	11

**Table 4:** Plackett–Burman design to evaluate factors affecting Taxol production by *A. flavus*

Run	X1	X2	X3	X4	X5	X6	X7	X8	X9	X10	X11	Taxol (µg/l)		
												Experimental	Predicted	Residuals
**1**	1	1	− 1	− 1	− 1	1	− 1	1	1	− 1	1	177.48	216.5	− 39.02
**2**	− 1	− 1	1	− 1	1	1	− 1	1	1	1	− 1	96.52	109.4	− 12.88
**3**	− 1	− 1	− 1	− 1	− 1	− 1	− 1	− 1	− 1	− 1	− 1	212.71	133.5	79.21
**4**	1	− 1	− 1	− 1	1	− 1	1	1	− 1	1	1	191.75	130.1	61.65
**5**	1	1	− 1	1	1	1	− 1	− 1	− 1	1	− 1	191.75	209.7	− 17.95
**6**	1	1	1	1	1	− 1	− 1	− 1	1	− 1	1	123.88	120.3	3.58
**7**	− 1	1	1	− 1	1	1	1	− 1	− 1	− 1	1	189.12	247.6	− 58.48
**8**	− 1	1	1	1	− 1	− 1	− 1	1	− 1	1	1	225.35	280.4	− 55.05
**9**	− 1	1	− 1	1	1	− 1	1	1	1	− 1	− 1	123.88	99.8	24.08
**10**	1	1	1	− 1	− 1	− 1	1	− 1	1	1	− 1	160.12	158.4	1.72
**11**	1	− 1	1	1	− 1	1	1	1	− 1	− 1	− 1	222.71	232.1	− 9.39
**12**	− 1	− 1	− 1	1	− 1	1	1	− 1	1	1	1	160.12	137.6	22.52

#### Optimization of Taxol Productivity by A. flavus with FCCD

The faced central composite design was adopted for further optimization studies using the significant variable that from the Plackett–Burman design. The results of 20 runs of three variables with positive effects demonstrated that Taxol productions diversified markedly with the condition were tested in the range of 96.5–302.7 µg/l. The maximum yield of Taxol by *A. flavus* (302.7 µg/l) was observed using cysteine (0.50 g/l) at pH 6.0 and incubated for 15 days as recorded in Table 5. Minimum Taxol production was recorded at a run number 15, where cysteine (1.0 g/L) at pH 7.0 and incubated for 7 days. The statistical analysis of the linear model design demonstrates that the F-value is 87.08 implying the significance of the model, and the values of “Prob > F” less than 0.050 indicate that model terms are significant (Table 6). Therefore, cysteine, pH, and incubation time are significant model terms. The analysis revealed that the “Pred. R-Squared” of 0.8972 is in reasonable agreement with the “Adj. R-Squared” of 0.9315. “Adeq. Precision” ratio of 30.800 measures the signal to noise ratio, as the ratio greater than four that being desirable, the Adeq. The results obtained from the CCD experiment were analyzed by ANOVA that yielded the following regression equation at the level of Taxol production: first-order model equation terms of coded factors — Taxol Conc. =  + 11.47 + 1.60A + 2.43B + 0.67C. The three-dimensional response surface curves showing the effect of interactions of the most significant parameters, cysteine, pH, and incubation time, were shown in Fig. 4.

**Table 5:** FCCD optimization design for the significant variables affecting Taxol production by *A. flavus*

Runs	E: cysteine	J: pH	K: incubation time (days)	Taxol yield (µg/l)		
				Experimental	Predicted	Residuals
1	0.75	6.50	9.50	189.04	189.62	− 0.58
2	0.75	7.34	9.50	122.48	142.0	− 19.52
3	0.75	6.50	9.50	189.04	189.62	− 0.58
4	0.75	6.50	9.50	189.04	189.62	− 0.58
5	1.00	7.00	7.00	133.23	123.88	9.35
6	0.75	6.50	13.70	169.71	175.07	− 5.36
7	1.0	6.00	7.00	212.37	219.12	− 6.75
8	0.50	6.00	12.00	244.86	255.35	− 10.49
9	1.17	6.50	9.50	142.39	147.45	− 5.06
10	0.75	6.50	9.50	189.04	189.62	− 0.58
11	1.00	7.00	7.00	110.25	96.52	13.73
12	0.75	6.50	9.50	189.04	189.62	− 0.58
13	0.75	6.50	5.30	208.36	203.3	5.06
14	0.75	6.50	9.50	189.04	189.62	− 0.58
15	0.50	6.00	12.00	**287.84**	**302.72**	− **14.88**
16	0.33	6.50	9.50	235.69	221.42	14.27
17	0.75	5.66	9.50	255.59	236.86	18.73
18	0.50	7.00	12.00	165.71	160.12	5.59
19	0.50	7.00	7.00	188.69	187.48	1.21
20	1.00	6.0	12.00	189.39	191.75	− 2.36

**Table 6:** ANOVA analysis for response surface linear model for the experiments with CCD

Source	Sum of squares	df	Mean square	F value	p-value Prob > F	Significance	R-squared	Std. dev
Model	121.60	3	40.53	87.08	< 0.0001	Significant	**0.9423**	**0.68**
E: cysteine	34.80	1	34.80	74.76	< 0.0001	Significant		
J: pH	80.62	1	80.62	173.21	< 0.0001	Significant		
K: incubation time (days)	6.18	1	6.18	13.28	0.0022	Significant		
Residual	7.45	16	0.47					
Lack of fit	6.92	11	0.63	6.00	0.0303	Significant		
Pure error	0.52	5	0.10					
Cor total	129.04	19						

### Impact of Gamma Radiation on A. flavus on the Taxol Productivity

The effect of gamma irradiation on *A. flavus* and on their Taxol yield was estimated by irradiating the fungal spores at different doses of γ-rays, then growing the spores on the modified malt extract medium obtained from the RSM by Plackett–Burman design. After cultural incubation under standard conditions, Taxol was extracted and quantified by TLC and HPLC. From the obtained results (Fig. S1), there is no significant effect on Taxol productivity upon γ-irradiation at the different doses.

### Synthesis of AuNPs and Conjugation with A. flavus Taxol Mediated by γ- Radiation

**Fig. 5:**
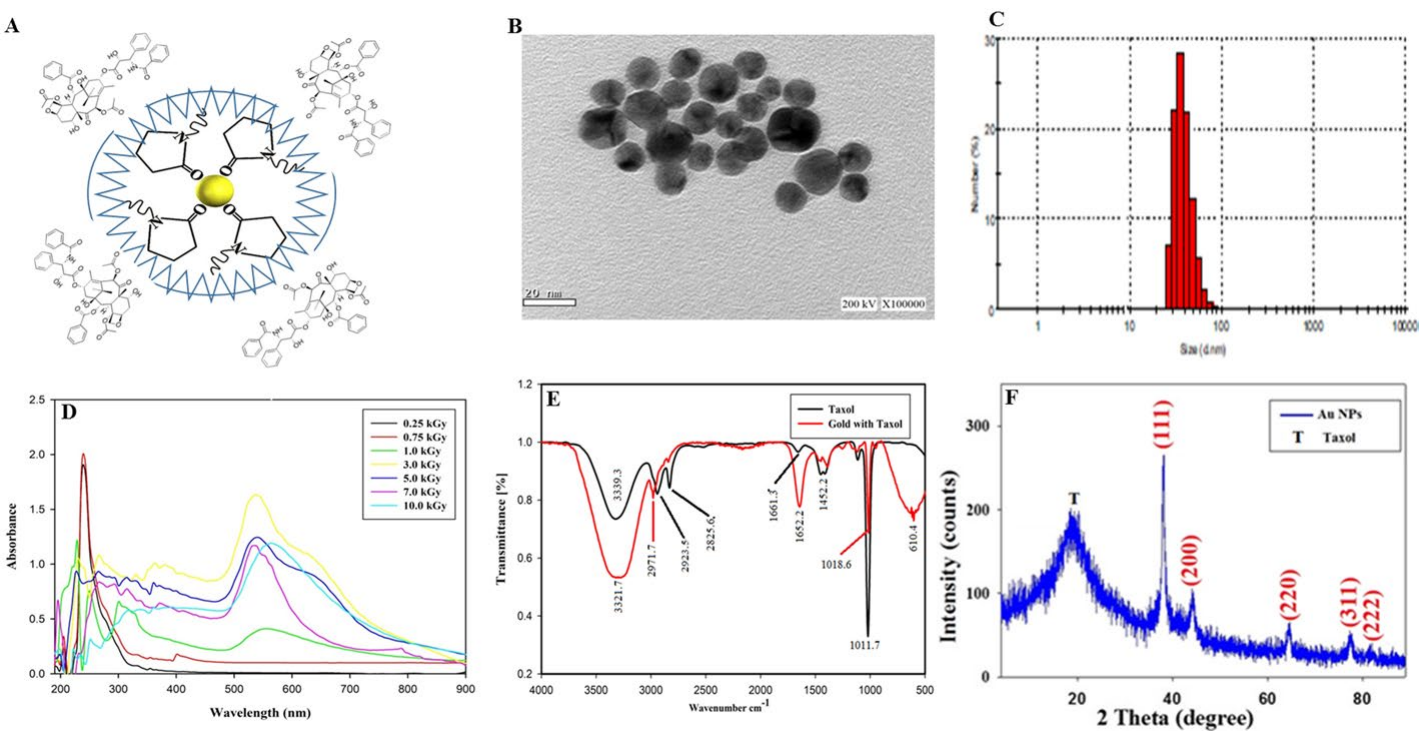
Physicochemical properties AuNPs-Taxol conjugates in response to different doses of γ-irradiation. **A** Scheme of Taxol conjugation with polyvinylrrolidone-gold nanoparticles, mediated by γ-irradiation at 1.0 kGy. **B** High resolution transmission electron microscope (HRTEM) of AuNPs. **C** Dynamic light scattering (DLS) pattern of the synthesized AuNPs. **D** UV–Vis spectrum of AuNPs at different doses of γ-irradiation. **E** FTIR spectra of Taxol and AuNPs-Taxol conjugates at 1.0 kGy. **F** X-ray diffraction (XRD) analysis of AuNPs and Taxol-AuNPs conjugates at 1.0 kG

Polyvinylpyrrolidone **(**PVP)-capped gold nanoparticles (AuNPs) were synthesized by mixing PVP with gold (III) chloride, mediated by gamma irradiation at doses of 0.25–10.0 kGy, and the developed PVP-AuNPs were evaluated from the UV–Vis, FT-IR, and XRD analyses. The sketch of formation of Taxol-PVP-AuNPs consortium was shown in Fig. 5. Size of the developed PVP-AuNPs mediated by 1.0 kGy gamma rays was monitored by DLS, and it was about 50 nm (Fig. 5). From the HRTEM image, the expected shape and size of the synthesized AuNPs was demonstrated with spherical particles within the nanoscale range from 20 to 30 nm with the average main diameter of 25 nm (Fig. 5). Similar results were observed with PVP-AuNPs mediated by gamma-irradiation [49, 50]. The spectral properties of the developed AuNPs in response to different doses of gamma rays were assessed from the UV–Vis analysis, with maximum recorded absorption spectrum at λ_540_ nm at 1.5–3.0 kGy. In addition, the development of AuNPs was monitored from the visual inspection, with a plausible yellowish to brown color, revealing the formation of AuNPs.

The chemical conjugation of Taxol and PVP-AuNPs was checked from the FT-IR spectral analysis, as revealed from the slightly shift of the intensity and transmission ratio of the functional groups of Taxol-PVP-AuNPs conjugates comparing to Taxol as control. The intensity of the peak at 3393.3 cm^−1^ assigned for the hydroxyl (OH) in Taxol-PVP-AuNPs consortium was increased by about 3 folds comparing native Taxol, revealing the chemical stretch on the hydroxyl groups (Fig. 5). As well as the intensity of the peaks 1661.0 cm^−1^ referring to C = O stretching frequency was strongly increased by about 10 folds upon conjugation with PVP-AuNPs, ensuring the stretching on the C = O bonds. The intensity of the peak 2923.5 corresponding to aliphatic CH stretching was quite stable. A slight shifting on the intensity of observed peaks at 1452.0 cm^−1^ and 1404.0 cm^−1^ has been observed due to the NH stretching frequency. The intensity of the peak 1109.0 cm^−1^ of carbonyl group-oxygen stretching frequency was slightly reduced upon conjugation with PVP-AuNPs.

The crystal/physical structure of the Taxol upon conjugation with PVP-AuNPs were resolved from the XRD analysis (Fig. 5). From the XRD, the Taxol-PVP-AuNPs consortium had the same structural configuration, crystal orientation, and size of the native-Taxol, ensuring the lack of negative effect on the crystal structure of native Taxol. From the results, the amorphous and crystal structure of the Taxol-PVP-AuNPs comparing to the native Taxol has been confirmed. From the XRD analysis, conjugation of Taxol and PVP-AuNPs has been resolved as revealed from the diffraction properties concerning 2ɵ = 38.18°, 44.01°, 64.57°, 77.67°, and 81.74° which described the Bragg’s observations at (111), (200), (220), (311), and (222), respectively. All the peaks were related to the ideal card of Joint Committee on Powder Diffraction Standards (JCPDS) of AuNPs (JCPDS card No. 04–0784) [51]. So, the strength of crystals of the synthesized AuNPs was shown, providing the face-centered cubic (fcc) crystalline structure. In addition, there is a simply amorphous peak at 19.25° for Taxol that is included in the organization and permanence of AuNPs. The XRD pattern confirmed the successful conjugation of Taxol and PVP-AuNPs. The mean crystallite size of the incorporated AuNPs was determined from the Scherrer’s equation [52], and it was 20.2 nm for Taxol-PVP-AuNPs consortium mediated by gamma rays as mentioned in equation:

$$\mathrm{D}=\frac{k\lambda }{\beta \mathrm{cos}\theta }$$

*where D is the average crystallite size, β is the full-width at half maximum, λ is the X-ray wavelength, and θ is the Bragg’s angle, *and* k is a constant.*

### Anticancer Activity of Taxol-PVP-AuNPs Conjugates

**Fig. 6:**
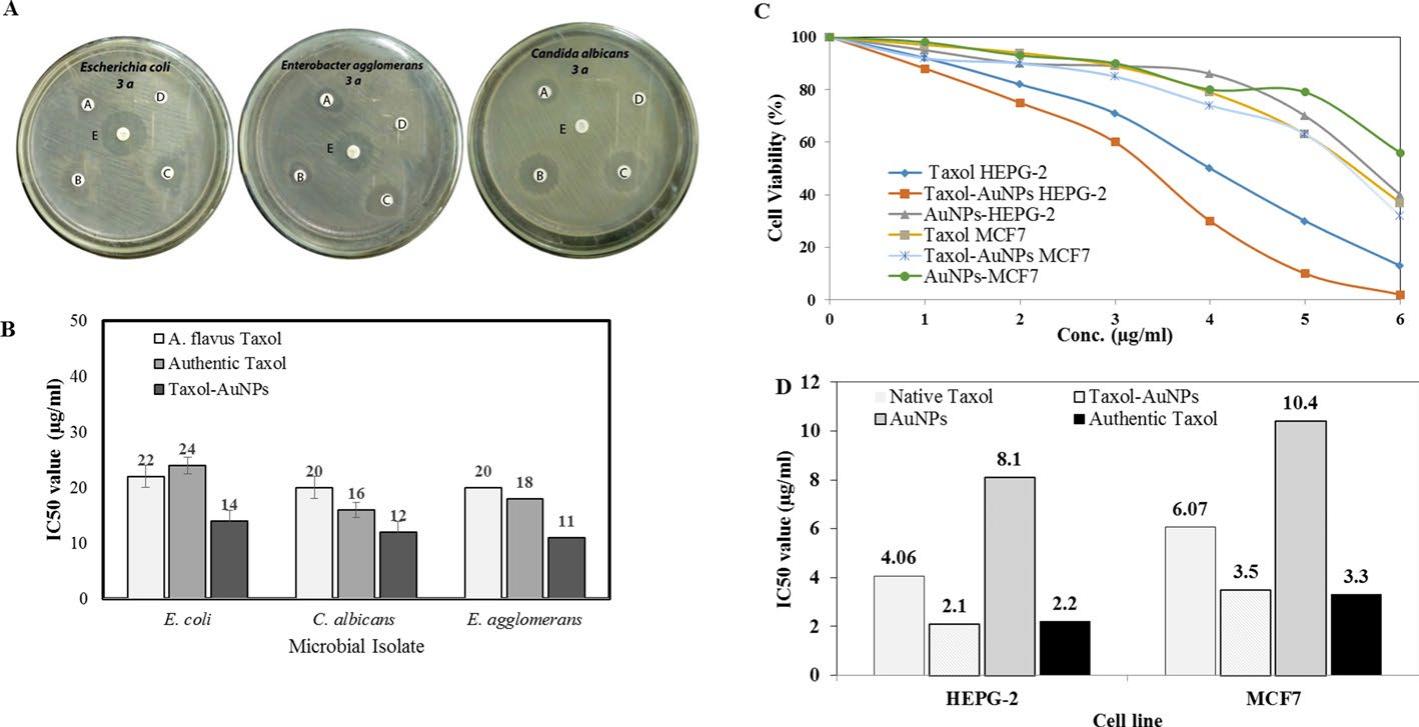
Antimicrobial and anticancer activities of the purified Taxol samples and Taxol-AuNPs conjugates. A Antimicrobial of native and Taxol-AuNPs conjugates towards *E. coli*, *E. agglomerans*, and *C. albicans* s activity, as revealed from the inhibition zones (A is the authentic Taxol, B is the *A. flavus* Taxol, C is the Taxol-AuNPs conjugates, D methanol, and E is the amoxicillin calvulanic acid). B The IC50 values of the authentic Taxol, native *A. flavus* Taxol, and Taxol-AuNPs conjugates. C Antiproliferative activity of native Taxol and Taxol-AuNPs conjugates towards HEPG-2 and MCF-7 cells as revealed from the cell viabilities. D The IC50 values of Taxol conjugates against the HEPG-2 and MCF-7 normalizing to authentic Taxol and AuNPs

The antiproliferative activity of Taxol-PVP-AuNPs conjugates was assessed against HEPG-2 and MCF-7 cell lines, normalizing to native Taxol and AuNPs, separately. From the results (Fig. 6), the bioactivity of Taxol was dramatically increased upon conjugation with AuNPs, comparing to native Taxol as control. As revealed from the IC_50_ values, Taxol-PVP-AuNPs consortium displayed the significant activity against HEPG-2 (2.2 µg/ml) and MCF-7 (3.3 µg/ml) comparing to native Taxol and AuNPs separately. So, upon conjugation with AuNPs, the activity of Taxol was increased by two folds towards the tested cell lines. Regarding to the bioactivity of AuNPs, the antiproliferative activity of Taxol-PVP-AuNPs consortium was increased by about 4 folds comparing to AuNPs as control. Interestingly, the activity of Taxol-AuNPs consortium was plausibly consistent with the authentic Taxol.

### Antimicrobial Activity Taxol and Synthesized AuNPs Against Different Microorganism

The antimicrobial activity of the extracted *A. flavus* Taxol and Taxol-AuNPs consortium was evaluated against various multidrug resistant bacteria and fungi (Table 7). Upon conjugation with AuNPs, the antimicrobial activity of Taxol has been strongly increased comparing to native Taxol and AuNPs separately, as revealed from the diameter of inhibition zones of the tested microorganisms (Fig. 6). As shown in Fig. 6, the Taxol-AuNPS consortium displayed the highest activity against *E. agglomeranus* (11 µg/ml), *C. albicans* (12 µg/ml), and *E. coli* (14 µg/ml), comparing to native *A. flavus* Taxol and authentic one. The higher activity of Taxol upon conjugation with AuNPs authenticates the bioavailability, solubility, and efficacy of Taxol to bind with the target tubulin protein in vivo.

**Table 7:** Antimicrobial activity of Taxol against different pathogenic microorganisms represented by diameter of inhibition zone (mm)

	**Authentic taxol**	**AMC**	**NS**	***A. flavus* Taxol**	**Taxol AuNPs**
*Bacillus subtilis* ATCC 6633	11.3 ± 0.5	9.0 ± 0.0	–-	14.0 ± 1.0	15.0 ± 1.0
*Pseudomonas aeruginosa*	8.6 ± 0.5	6.0 ± 0.0	–-	11.3 ± 1.1	10.0 ± 1.0
*Escherichia coli*	9.3 ± 0.5	23.3 ± 0.57	–-	17.0 ± 1.0	16.0 ± 1.1
*Staphylococcus epidermidis*	8.6 ± 0.5	25.0 ± 1.0	–-	13.6 ± 1.5	11.0 ± 1.0
*Staphylococcus aureus*	9.3 ± 1.5	24.6 ± 1.5	–-	12.0 ± 1.0	12.0 ± 1.0
*Enterobacter agglomerans*	12.0 ± 0.0	20.0 ± 0.0	–-	13.0 ± 2.0	17.0 ± 2.0
*Klebsiella pneumoniae*	9.6 ± 0.5	6.0 ± 0.0	–-	9.0 ± 1.0	9.0 ± 1.0
*Candida albicans*	11.6 ± 0.5	––	6.0 ± 0.0	24.3 ± 1.5	25.0 ± 1.5

The concentration of authentic Taxol and *A. flavus* Taxol was 20 µM

## Discussion

Fungal endophytes with Taxol producing potency raised the hope for mass production of Taxol due to their fast growth, cost effective fermentation process, independence on climatic changes, and feasibility of genetic manipulation. However, the anticipation of fungi for industrial production of Taxol has been challenged by their lower reproducible yield and loss of Taxol productivity with the subculturing [14, 16, 26, 39, 45, 53, 54]. Most of the endophytic Taxol-producing fungi were isolated from *Taxus* sp. and *Podocarpus* sp. which are belonging to family Taxaceae [18, 39]. Exploring of the Taxol producing by endophytic fungi from plants outside Taxaceae family with probable Taxol productivity is the main objective by biotechnologists. *Jojoba* plant is one of the most traditionally recognized medicinal plants for its ethnopharmacological relevance such as antimicrobial activity, anti-inflammatory, and anticancer activities [24, 55]. Thus, the objective of this study was to isolate and estimate the Taxol producing potency of the endophytic fungi from the *jojoba* plant. Among the recovered fungi, the endophytic isolate *A. flavus* gave the highest Taxol yield (88.65 μg/l), as authenticated from the TLC, UV- absorption, and HPLC analysis. Similar screening paradigm for Taxol production has been reported for endophytes from *Ginko biloba* [28] and other plants [15, 18, 20, 39, 56]. The Taxol yield by *A. flavus* has been agreed with *P. polonicum*, an endophytes of *Ginkgo biloba* [28] and *A. flavipes* [15, 39], *A. terreus* [15, 39], an endophytes of *P. gracilior*, as well as for endophytes of *Taxus* spp. such as *A. candidus*, *Fusarium solani* [57], *A. niger* [58], and *A. fumigatus*. The identity of *A. flavus*, the potent Taxol producing fungal isolate, was confirmed from the molecular analysis of the ITS region, and the sequence was deposited on Genbank with accession #MW485934.1, as well as at Assiut University Mycological Center (AUMC), Egypt with deposition # AUMC13892. Similarly, the identity of *A. flavus* was confirmed based on the sequence of ITS region [49, 59], 2018, 2019, [1, 11, 36, 43, 60–68]. The chemical structure of extracted Taxol from *A. flavus* was ^1^HNMR, and FT-IR analyses. The resolved signals of HNMR for *A. flavus* Taxol were identical to the standard Taxol, which distributed between 1.0 and 8.0 ppm. Three proton signals were resolved at 1.0–3.0 ppm corresponding to methyl, acetate, and acetylene groups, whereas the signals for aromatic moieties were resolved at 6.5–9.0 ppm [17, 45]. Consistently, for all Taxane scaffolds, signals for their side chains protons were resolved at 2.0–7.0 ppm, while those for benzoate (C2), phenyl (C3), and benzamide (C3) groups were resolved at 7.0 and 8.4 ppm [69] ( [70]. The FT-IR spectra of *A. flavus* Taxol were like authentic Taxol, as coincident with Taxol from other fungal isolates [3], Visalakchi et al. 2010). The yield of Taxol from *A. flavus* was optimized by response surface methodology using Plackett–Burman Design [17–20, 45, 46, 49, 71]. The yield of Taxol was increased by about 1.8 folds upon on response to factorial design nutritional optimization process [17, 45]. The highly significant variables affecting Taxol production by *A. flavus* was further optimized with the CCD design, giving the maximum yield (302.72 μg/l) with cysteine (0.5 g/L) at pH 6.0 and incubated for 7 days. In an endeavor to enhance the production of Taxol by *A. flavus*, the fungal spores were exposed to ionizing γ- radiation, and the spores were grown on the modified malt extract broth medium, and Taxol was extracted and quantified. Upon γ-rays irradiation, the Taxol yield by *A. flavus* was not significantly increased comparing to the control cultures, suggesting the lack of induction of biosynthetic gene cluster of Taxol. However, the Taxol yield was slightly increased by *Fusarium maire* and *Nodulisporium sylviforme* in response to gamma-irradiation [1, 72], El-Sayed et al., 2020). Taxol productivity by *A. flavus* in response to different media has been studied. *A. flavus* gave the highest Taxol yield by growing on Czapek’s-Dox media and malt extract, as consistent with results for Taxol production by *A. terreus* [16, 53] and *P. polonicum* [28]. However, preference of PDB for Taxol production was reported for *A. candidus* and *F. solani* [73].

To increase the targetability, solubility, and efficacy of Taxol to bind with the target protein in vivo, several successful trails have been motivated. Conjugation of AuNPs with the less soluble chemotherapeutic drugs is one of the recent magnificent technologies to increase the solubility and targetability of several chemotherapeutic drugs. The purified Taxol from *A. flavus* was conjugated with AuNPs, in the presence of PVP as capping and stabilizing agent, mediated by irradiation by γ-rays. The AuNP was prepared by γ-irradiation-based reduction in the presence of PVP as stabilizing agents to prevent the metal colloids from the rapid aggregation, as revealed from the color change and a strong absorption band at λ_540_ nm. The color change is usually attributed to the surface plasmon resonance (SPR) [74], and the sharp and high intensity peaks ensure the highest yield and size distribution of the synthesized AuNPs. Gamma-irradiation has been authenticated as one of the most desirable methods for synthesis of metallic nanoparticles due to their highly reducing radicals and generating free electron without byproduct formation [50]. The particle size obtained from DLS measurements 47.58 nm was larger than the TEM results (13.0–21.0 nm) because DLS analysis measures the hydrodynamic radius. From the XRD analysis, the development of crystal structure of Taxol-PVP-AuNPs consortium has been as revealed from the diffraction peaks at 2ɵ = 38.18°, 44.01°, 64.57°, 77.67°, and 81.74° which described the Bragg’s observations at (111), (200), (220), (311), and (222), respectively [51]. The synthesized AuNPs showed crystal strength and provided the face-centered cubic (fcc) crystalline structure, with one amorphous peak at 19.25° for Taxol that is included in the organization and permanence of AuNPs [52].

The antiproliferative activity of Taxol-PVP-AuNPs conjugates was assessed against HEPG-2 and MCF-7 cell lines, normalizing to native Taxol and AuNPs, separately. The bioactivity of Taxol was dramatically increased upon conjugation with AuNPs, comparing to native Taxol as control.

The enhanced anticancer activity of Taxol by AuNPs could be due to the decreasing on the lymph drainage, increasing the blood circulation and Taxol solubility upon conjugation with AuNPs, and comparing to native Taxol [75]. AuNPs receive a great attention due to their low toxicity, higher versatility to bind to with different molecules, and biocompatibility [76]. From the IC_50_ values, Taxol-PVP-AuNPs consortium displayed the significant activity against HEPG-2 and MCF-7, comparing to native Taxol and AuNPs separately. Regarding to the bioactivity of AuNPs, the antiproliferative activity of Taxol-PVP-AuNPs consortium was increased by about 4 folds comparing to AuNPs as control. Interestingly, the activity of Taxol-AuNPs consortium was plausibly consistent with the authentic Taxol. The antimicrobial activity of *A. flavus* Taxol-AuNPs consortium was evaluated against various pathogenic multidrug resistant microorganisms. Upon conjugation with AuNPs, the antimicrobial activity of Taxol was strongly increased comparing to native Taxol and AuNPs, separately. Taxol-AuNPS consortium displayed the highest activity against *E. agglomeranus*, *C. albicans*, and *E. coli.* The higher activity of Taxol upon conjugation with AuNPs authenticates the bioavailability, solubility, and efficacy of Taxol to bind with the target tubulin protein in vivo.

In conclusion, *Aspergillus flavus* MW485934.1, an endophyte of jojoba, has been isolated and identified as potent Taxol producer, based on the metabolic and chromatographic analysis. The chemical identity of extracted Taxol from *A. flavus* was verified from the TLC, HPLC, NMR, and FTIR analyses. The yield of Taxol by *A. flavus* was maximized by the response surface methodology with the Plackett–Burman and faced central composite designs. Upon using factorial designs RSM, the yield of Taxol by *A. flavus* was increased about 3.2 folds (302.7 µg/l), comparing to control cultures (96.5 µg/l). In addition, conjugates of Taxol-gold nanoparticles (AuNPs) mediated by γ-rays were prepared. The physical and spectroscopic properties of the Taxol-AuNPs conjugates were determined by UV–Vis, dynamic light scattering (DLS), X-ray diffractometer (XRD), and transmission electron microscope (TEM) analyses. With AuNPs conjugation, the anticancer activity towards different tumor cell lines was dramatically increased. As well as the antimicrobial activity of Taxol-AuNPs conjugates towards the different multidrug resistant bacteria was strongly increased comparing to native Taxol compounds.

## Supplementary Information

Below is the link to the electronic supplementary material.

Supplementary file1 (TIF 61 KB)

## Data Availability

All the data are available on this manuscript.
