# Supplementary figures and images for: Boosting the Anticancer Activity of Aspergillus flavus “endophyte of Jojoba” Taxol via Conjugation with Gold Nanoparticles Mediated by γ-Irradiation
Source: Appl Biochem Biotechnol. 2022 Apr 19;194(8):3558–81. doi: 10.1007/s12010-022-03906-8 (PMC9270289; doi:10.1007/s12010-022-03906-8)

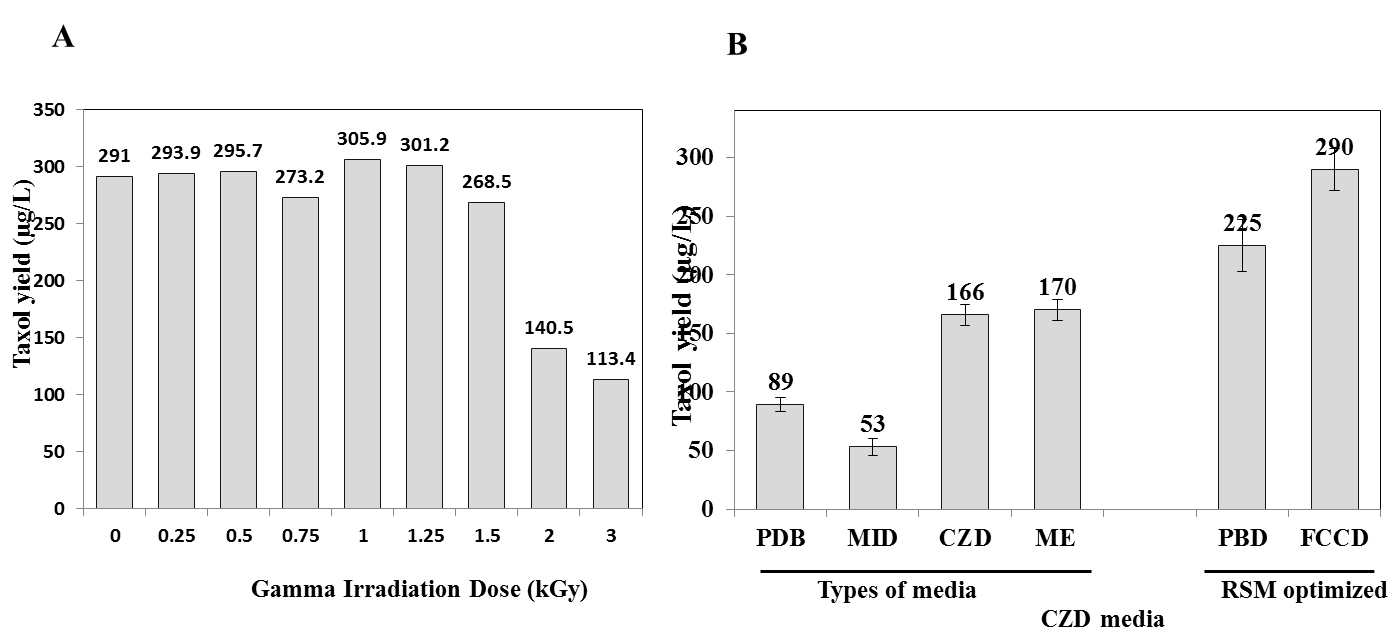

Supplement: Supplementary file 1 — Supplementary file1 (TIF 61 KB) [file 12010_2022_3906_MOESM1_ESM.tif]
